# Cognitive dissonance in infertility treatment: Why is it so difficult to discard disproven therapies, like the endometrial scratch?

**DOI:** 10.1016/j.xfre.2022.05.005

**Published:** 2022-05-18

**Authors:** Richard J. Paulson

**Affiliations:** Division of Reproductive Endocrinology and Infertility, USC-HRC Fertility, Pasadena, California

At a recent fertility meeting, at yet another lecture on “Recurrent Implantation Failure,” I heard the presenter suggest that perhaps the endometrial scratch procedure has some benefit for patients who have failed multiple embryo transfer attempts. I was surprised that in 2022, someone would think that this thoroughly disproven therapy should still be considered as “potentially useful.” However, I continue to see patients who have been advised to have this treatment, suggesting that this is not an isolated sentiment.

The therapeutic intervention of the endometrial scratch is not alone. Rather, it is just one example of the numerous therapies that have made their way into the practice of reproductive endocrinology and infertility on the basis of tenuous data. Our field is cluttered with unproven supplements, questionable evaluations, and dubious therapies. Evidence-based treatments need to be based on well-designed studies, but our field seems to work backward: in an effort to remain at the “cutting edge,” therapies are often adopted as “potentially useful” on the basis of retrospective data (1) and then only later evaluated with larger trials. Because such follow-up investigations are rarely definitive, treatments remain in common practice for several years without any definitive proof that they are efficacious. In some cases, like that of the endometrial scratch, they may even be disproven by randomized controlled trials (2) and yet continue to be advised to patients. This phenomenon must be interpreted as cognitive dissonance. Data that contradict a commonly held worldview are either ignored (confirmation bias) or rationalized (e.g., by pointing out that the randomized controlled trial did not include all potential subgroups of patients and, therefore, is not definitive.)

From the patient perspective, this is not a trivial problem. If a practitioner presents a patient with a therapy that is “potentially useful,” that patient may, consciously or subconsciously, attribute a numerical value to the potential benefit. For example, if the perceived “potential benefit” of a given therapy is 10%, then it seems reasonable to invest $2,000 to improve the outcome of a $20,000 fertility treatment. The trouble is that there are dozens of “potentially useful” interventions, and those numbers simply do not add up.

The obligation of those of us who are specialists in this field is to draw a sharp distinction between proven, unproven, and disproven therapies. Let us begin by discarding treatments that have been disproven and focus on giving patients clear information about what has been demonstrated to have efficacy and what has not. There is already a robust market for unproven supplements, vitamins, and diets (3) that do little other than add cost to treatment. Let us begin by letting patients know that these are not evidence-based. Furthermore, let us finally stop advocating for the endometrial scratch (4, 5).
